# Nanoparticle-delivered immunomodulators targeting blood–brain barrier disruption in fulminant adult-onset ADEM

**DOI:** 10.1097/MS9.0000000000005089

**Published:** 2026-04-30

**Authors:** Eraj Nadeem, Sabeen Tahir, Waqar Ahmed Cheema, Muhammad Talha, Mahnoor Fatima

**Affiliations:** a Department of Medicine, United Medical and Dental College, Karachi, Pakistan; b Department of Medicine, Federal Medical College, Islamabad, Pakistan; c Department of Medicine, Independent Medical College, Faisalabad, Pakistan; d Department of Medicine, King Edward Medical University, Lahore, Pakistan; e Department of Medicine, Shaheed Ziaur Rahman Medical College, Rajshahi Medical University, Bogura, Bangladesh

*Dear Editor,*

Acute Disseminated Encephalomyelitis (ADEM) is an autoimmune, inflammatory condition characterized by demyelination of the central nervous system (CNS) secondary to acute inflammation. Approximately 80% of the patients exhibit multifocal neurological deficits, and nearly half report a preceding viral illness or immunization. Although ADEM is primarily a disease targeting children, new research reports the rising prevalence of ADEM in adults manifesting without conventional triggers. In adults, fulminant forms of ADEM are particularly severe, and the manifestations are exacerbated by the inflammatory cascade producing cytokines, which increase vascular permeability and break down the blood-brain barrier (BBB). Traditional immunomodulatory and immunosuppressive therapies are associated with systemic side effects like increased infection risk and drug toxicity; additionally, these methods are only partially effective^[^1^]^.

Recent advancements suggest a novel therapy involving nanoparticles like liposomes, polymeric nanoparticles, and exosome-based carriers, which can be engineered to cross the BBB and selectively deliver pharmacological agents to target sites. Immunomodulatory agents like interferon-beta and glatiremer acetate suppress CNS inflammation by limiting T-cell migration across the BBB, suppressing pro-inflammatory cytokine migration, and promoting immune tolerance. Targeted delivery may enhance interparenchymal drug concentrations at sites of neuroinflammation, improving efficiency and limiting systemic immunosuppression^[^2^]^. Preclinical demyelinating models treated by nanoparticle-delivered immunomodulators show up to 70% decrease in peak clinical scores compared to soluble treatments, which show promising clinical translational potential for immunomodulation therapy in demyelinating diseases such as multiple sclerosis (MS) and ADEM^[^3^]^.

Recent research underscores engineered nanoplatforms for effective blood-brain-barrier penetration. Nanoparticles with a surface area of 20 nm are promising vehicles for targeted CNS intervention. Strategic surface functionalization with targeted ligands further promotes receptor-mediated transcytosis, thereby optimizing site-specific drug delivery^[^4^]^. Mechanistic insights into blood-brain-barrier transport further clarify the application of nanoparticles; nearly 98% of small-molecule drugs and 100% of large biologics fail to reach therapeutically effective concentrations within the brain. Consequently, polymeric nanoparticles, maintained below 200 nm, have demonstrated improved endothelial uptake and enhanced intracerebral distribution^[^5^]^.

Nanoparticle-based therapies are significantly effective, but they face several challenges. BBB transport remains highly selective, with efficacy dependent on nanoparticle size, shape, and surface chemistry. Rapid clearance by the liver and spleen, disease-associated alterations in BBB integrity, and manufacturing variability further complicate clinical translation. Additionally, the presence of efflux transporters further restricts nanoparticle penetration across the BBB. Moreover, limitations in current imaging modalities hinder the precise quantification of nanoparticle target engagement *in vivo*^[^6^]^.

The current understanding of adult-onset ADEM needs to develop new treatment methods because existing immunosuppressive therapies do not meet its requirements. Researchers need to examine targeted nanocarrier-based strategies for inflammatory demyelinating disorders because blood–brain barrier dynamics and conventional therapies restrict their ability to reach the central nervous system^[^5^]^. The development of new platforms that provide targeted immunomodulation capabilities supports the growth of nanomedicine research for neuroinflammatory disorders^[^3^]^. The current technological advancements require researchers to establish better collaborative frameworks between neuroimmunology and nanotechnology while developing efficient methods to transport nanoparticles across the blood-brain barrier and customize delivery systems for specific diseases^[^4^]^. Researchers need to develop better preclinical demyelinating models and fast-track clinical research projects, which need to validate both safety measures and treatment effectiveness for ADEM and related conditions^[^2,6^]^.

In conclusion, Nano-carrier systems do face challenges. However, careful optimization of size, shape, and surface properties, along with the use of protective coatings, can enhance transport efficiency, making them a promising platform for targeted brain treatment.

## Data Availability

Not applicable.
